# Graphdiyne Saturable Absorber for Passively Q-Switched Ho^3+^-Doped Laser

**DOI:** 10.3390/nano10091848

**Published:** 2020-09-16

**Authors:** Cheng Zhang, Qianqian Hao, Yuqian Zu, Mengyu Zong, Jia Guo, Feng Zhang, Yanqi Ge, Jie Liu

**Affiliations:** 1 Shandong Provincial Engineering and Technical Center of Light Manipulations and Shandong Provincial Key Laboratory of Optics and Photonic Device, School of Physics and Electronics, Shandong Normal University, Jinan 250358, China; 2018010053@stu.sdnu.edu.cn (C.Z.); 2019010057@stu.sdnu.edu.cn (Q.H.); 2018010055@stu.sdnu.edu.cn (Y.Z.); 2020010070@stu.sdnu.edu.cn (M.Z.); 2 Institute of Microscale Optoelectronics, Collaborative Innovation Center for Optoelectronic Science & Technology, Key Laboratory of Optoelectronic Devices and Systems of Ministry of Education and Guangdong Province, College of Physics and Optoelectronic Engineering, Shenzhen Key Laboratory of Micro-Nano Photonic Information Technology, Guangdong Laboratory of Artificial Intelligence and Digital Economy (SZ), Shenzhen University, Shenzhen 518060, China; guojia2018@email.szu.edu.cn (J.G.); zhangfeng01@szu.edu.cn (F.Z.); 3 Collaborative Innovation Center of Light Manipulations and Applications, Shandong Normal University, Jinan 250358, China

**Keywords:** graphdiyne nanomaterial, saturable absorber, passively Q-switched, Ho-doped laser, 2.1 μm

## Abstract

High-quality all-carbon nanostructure graphdiyne (GDY) saturable absorber was successfully fabricated and saturable absorption properties in the 2 μm region were characterized using a commercial mode-locked laser as a pulsed source. The fabricated GDY was first used as an optical switcher in a passively Q-switched Ho laser. Under absorbed pump power of 2.4 W, the maximum average output power and shortest pulse width were 443 mW and 1.38 µs, at a repetition rate of 29.72 kHz. The results suggest that GDY nanomaterial is a promising candidate as an optical modulator for generation of short pulses in Ho-doped lasers at 2.1 μm.

## 1. Introduction

Graphdiyne (GDY) is an emerging synthetic material with all-carbon nanostructure, following the footsteps of fullerenes (zero-dimensional, 1985), carbon nanotubes (one-dimensional, 1991), and graphene (two-dimensional, 2004) [1,2,3,4,5,6]. GDY, has attracted widespread attention with its unique electrical and optical properties. After theoretical chemist R. H. Baughman’s first proposal that the structure of GDY formed by sp and sp^2^ hybrid carbon could exist stably, many attempts to fabricate it have been made by researchers [7,8]. Until 2010, Li et al. were the first to successfully prepare large area GDY film on the surface of copper through the chemical in-situ growth of hexaethynylbenzene, realizing the leap from theoretical prediction to experimental testing of GDY [9,10,11]. GDY is a two-dimensional (2D) planar structure formed by the direct connection of sp-hybridized 1,3-diyne bonds and sp^2^-hybridized benzene rings. This planar hybridization structure gives GDY a high π conjugation and controllable electronic structure [12,13,14]. In the GDY structure model, alkyne bonds and sub-nanopores give a large amount of reaction sites for its functionalization. The special electronic structure and pore structure of GDY offer important potential applications in the fields of optoelectronics, electronics, energy, and catalysis [15]. In particular, GDY has a tunable direct band gap of 0.4–1.22 eV, in contrast with the zero-band gap of graphene, which shows great potential for application in photonic devices based on optical switching [16,17,18].

Optical switchers play a crucial role in pulsed laser operation. The Ho-doped pulsed laser sources at 2.1 µm possess a broad range of application in scientific and technical fields, such as laser radar, countermeasure, and remote sensing [19,20,21]. What is more, the Ho-doped pulsed laser has become a research hotspot owing to the features of low heat and high efficiency [22,23,24]. Passively Q-switched (PQS) technology is the most efficient approach to achieving pulsed lasers. In recent years, the emergence of two-dimensional (2D) materials has provided more choices and opportunities for the use of saturable absorbers (SAs) in pulsed lasers PQS solid-state lasers. They have common remarkable merits, including simple structure, compactness, easy operation, and low cost [25,26,27,28,29,30,31,32,33]. However, they also have their own disadvantages. For example: Carbon nanotubes (CNTs) easily form a bundled and entangled morphology, which causes strong scattering and thus large losses; thermal stability and oxidation resistance of black phosphorus (BP) and transition metal oxides (TMDs) have strongly limited their applications in ultrafast lasers; low absorption efficiency of graphene leads to low modulation depth, and so on [34,35,36]. GDY has a natural band gap energy compared to graphene and MXene with zero band gap. The large nonlinear absorption coefficient (>10^−1^ cm GW^−1^), low saturation intensity (<10 GW cm^−2^), and ultrafast relaxation time (<30 ps) have all confirmed that GDY as an SA is a promising 2D material in pulsed laser generation. Recently, Guo et al. reported the saturable absorption properties of GDY for the first time and used them to generate mode-locked fiber lasers at wavebands of 1 µm and 1.5 µm [37,38]. In 2020, Zu et al. reported GDY as a saturable absorber for 1.9 μm all solid-state Q-switched laser [39]. However, up to now there has been no pulsed laser performance reported based on GDY in a Ho-doped laser around 2.1 μm.

In this paper, we developed a GDY optical switcher with remarkable thermal stability, using it first as an SA at 2.1 μm region. In a compact linear cavity, we obtained stable pulsed Ho laser operation. Furthermore, the Ho-doped PQS laser characteristics were displayed from all sides. The research demonstrates that GDY nanomaterial is an expected material as an optical modulator in mid-infrared pulsed lasers

## 2. Fabrication and Characteristics of GDY-SA

**Figure 1:**
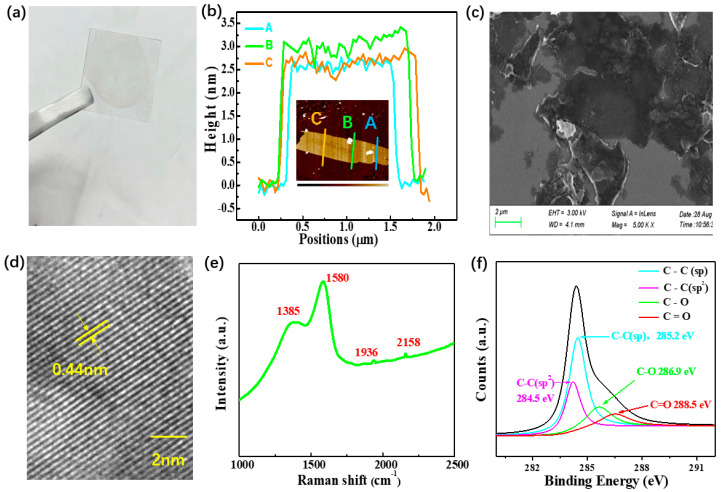
(**a**) Fabricated graphdiyne (GDY) sample on a glass substrate (10 × 10 mm^2^), (**b**) typical height profiles, inset: Atomic force microscopy (AFM) image, (**c**) SEM image, (**d**) high-resolution transmission electron microscope (HRTEM) image of GDY, (**e**) Raman spectrum of GDY, (**f**) high-resolution XPS spectrum of GDY.

The GDY material was first synthesized using the liquid/liquid interface method [40]. Subsequently, ethanol solution was added to the prepared GDY solution and the mixture was stirred ultrasonically for about half an hour. Next, we dropped solution onto the center of the quartz plate and centrifuge twice at a speed of 150 r/min to make the plate evenly covered. Finally, the GDY-SA used in our experiment was successfully prepared, as shown in Figure 1a. Its color is transparent and light brown, indicating good light transmittance. The illustration in Figure 1b shows the atomic force microscopy (AFM) (Bruker, Dimension ICON, Karlsruhe, Germany) image of GDY nanosheets. From the cross-section analysis we know the thickness of the GDY nanosheets is about 2.8 nm, as shown in Figure 1b, indicating the prepared sample is only a few layers thick. The thickness affects the modulation depth of the GDY-SA. The morphology characteristics of GDY nanosheets were detected using scanning electron microscope (SEM) (Sigma 500, ZEISS, Oberkochen, Germany). Figure 1c shows several microns in size, which displays that GDY nanosheets are easily folded during the process of drying before SEM measurement, due to the thin structural feature. As shown in Figure 1d, a great quantity of GDY nanosheets can be observed through high-resolution transmission electron microscope (HRTEM) (Talos F200X G2, FEI, Waltham, MA, USA) images, indicating that 2D GDY has been successfully fabricated. Clear lattice spacing of 0.44 nm was observed corresponding to the characteristics of GDY. Raman spectroscopy based on light scattering effect is an effective method to characterize the structure of carbon materials. It can qualitatively identify the order degree of samples and detect the existence of acetylenic linkages. Figure 1e shows the four typical Raman peaks of GDY sample, which are located at 1385 cm^−1^ (D bond), 1580 cm^−1^ (G bond), 1936 cm^−1^, and 2158 cm^−1^, respectively. The intensity ratio between D bond and G bond is about 0.73, indicating that the graphitic sample has a good degree of order. Peaks at 1936 cm^−1^ and 2158 cm^−1^ can be classified as characteristic peaks of acetylenic linkages. The high-resolution X-ray photoelectron spectroscopy (XPS) (Thermo Scientific ESCALAB 250Xi, Thermo Scientific, Waltham, MA, USA) (Figure 1f) confirmed the composition of GDY. The appearance of O1s peak is mainly caused by the small amount of air absorbed by GDY exposed to the air. The two C1s peaks at 285.2 and 284.5 eV are the sp orbital and sp^2^ orbital of the C atom. Furthermore, the integral area ratio of these two peaks is 2:1, which also indirectly indicates that the benzene rings are connected by two alkyne bonds.

**Figure 2:**
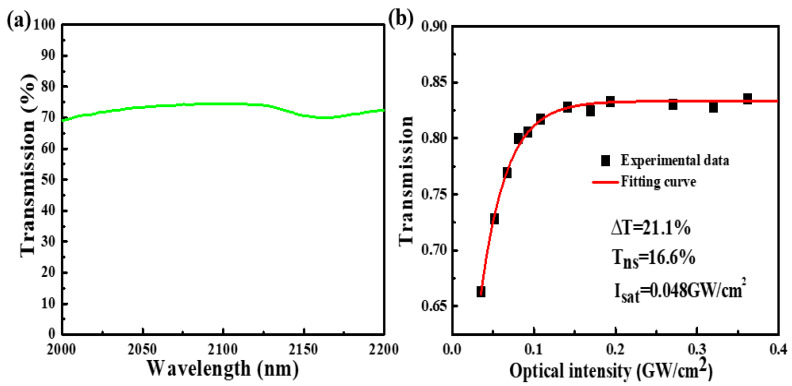
(**a**) Transmission spectrum, (**b**) nonlinear transmission curve.

Additionally, the transmission of the GDY-SA was measured to be higher than 70% in the 2000–2150 nm spectral range, demonstrating that the GDY-SA has potential for broadband optical modulation, as shown in Figure 2a. We also measured the nonlinear optical response of the GDY-SAs using a mode-locked Tm-doped fiber laser (MCL-2000-50-SM-20-PS, Mchlight, Shenzhen, China) at wavelength of 2000 nm with balanced synchronous dual-detector [41]. The transmittance was detected by varying intensity of the laser seed source power. We quoted a model of T (I) = 1 − ∆T × exp (−I/I_sat_) − T_ns_ to characterize nonlinear optical parameters (T (I), ∆T, I, I_sat_, and T_ns_ correspond to transmission, modulation depth, input optical intensity, saturation optical intensity, and non-saturable loss, respectively). By fitting the experimental results, the saturation optical intensity, non-saturable loss, and modulation depth were obtained as 0.048 GW/cm^2^, 16.6%, and 21.1%, respectively (Figure 2b). The appropriate modulation depth value and the low I_sat_ value in 2 000 nm band mean that GDY has great technical potential as a passive Q-switcher for generating ultrashort laser pulses.

## 3. Experimental Setup

**Figure 3:**
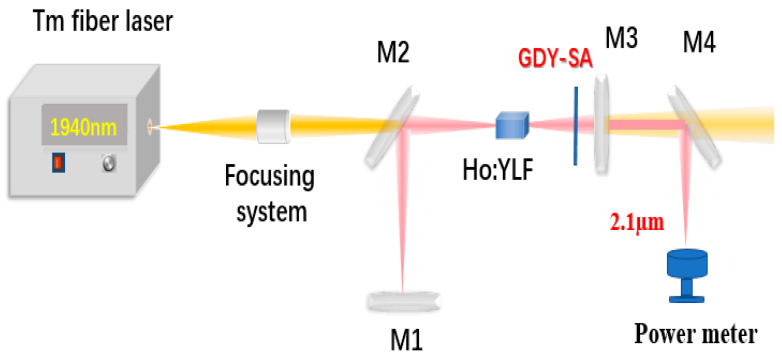
The schematic of the experimental setup for the passively Q-switched (PQS) operation.

A schematic diagram for the GDY-SA pulsed laser is shown in Figure 3. The pump source used in our experiment was a commercial Tm: Fiber laser (TDFL01-00015, CETC-Txstar, Shanghai, China) with a center wavelength of 1940 nm at 22 °C. A lens with a focal length of 100 mm was used as a coupling system to collimate and focus the pump laser into the crystal. The block shaped Ho:YLF crystal has a dopant concentration of 0.5 at%, with dimensions of 3 × 3 × 10 mm^3^. Both end faces are antireflection coated at 1940 and 2050 nm. In order to eliminate the heat generated during pumping process, the Ho:YLF crystal was wrapped in indium foil and tightly installed in a water-cooled copper block at a temperature of 13 °C. A simple linear resonator cavity was used to investigate the output performance of the Ho:YLF laser. An input mirror with a radius of curvature of 200 mm was high-transmittance (HT) at 1.9 μm and high-reflection (HR) at 2.1 μm. The 45-deg dichroic mirrors M2 and M4 were high transmission for pump light and high reflective for resonant wavelength. The plat mirror M3, serving as an output coupler (OC), gave partial transmissions (T) of 1% and 3% at 2.1 μm, respectively.

## 4. Results and Discussions

**Figure 4:**
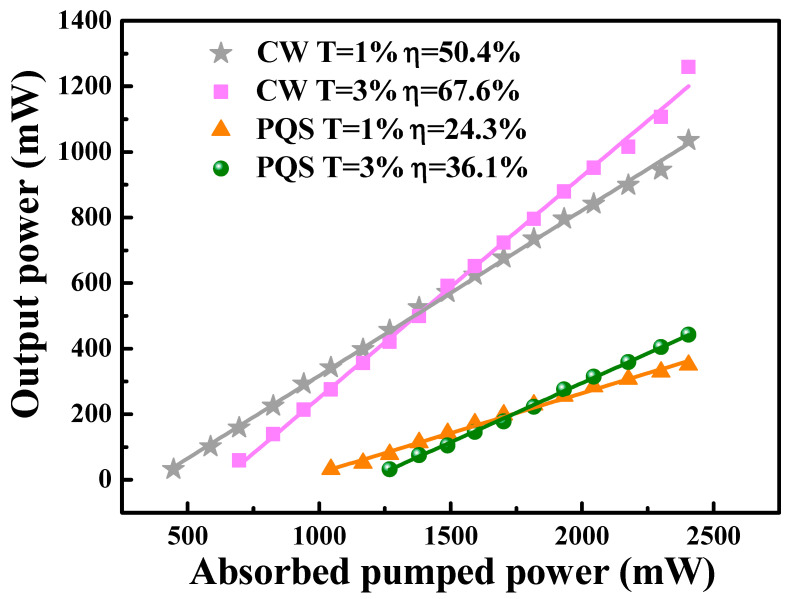
Output power versus the absorbed pump power for continuous-wave (CW) and PQS.

At the beginning, the performances of the continuous-wave (CW) laser was analyzed, as shown in Figure 4. The output the power was measured by a 30 A-SH-V1 (30ASH-V1, OPHIR, Jerusalem, Israel) laser power meter. Under the absorbed pump power of 2.4 W, the CW mode maximum output power for OCs of T = 1% and T = 3% were 1.04 W and 1.26 W, corresponding to slope efficiencies of 50.4% and 67.6%, respectively.

**Figure 5:**
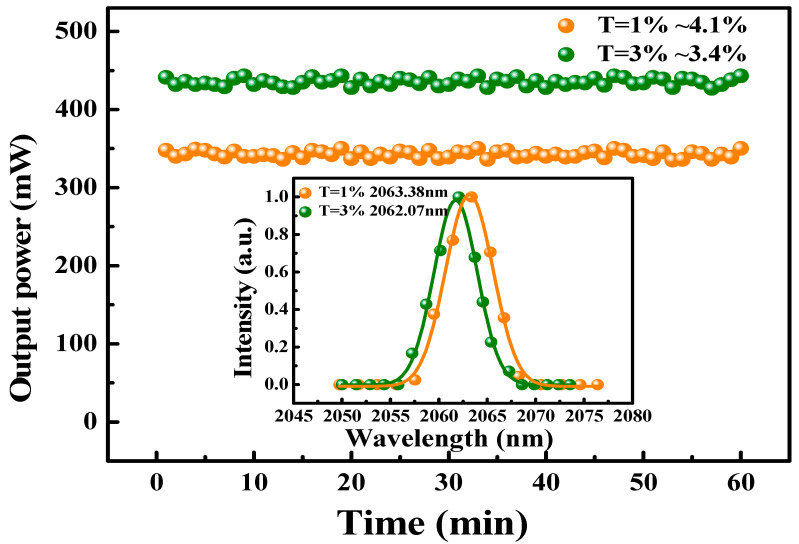
Instability of the average output power measured for 60 min with output couplers (OCs), inset: PQS laser emission spectra for two different OCs.

Subsequently, on installing the GDY-SA into the laser cavity at a 10 mm distance from the OC, stable PQS Ho:YLF laser mode was easily carried out. The beam radius of the Ho:YLF laser inside the resonator was calculated by using the ABCD matrix, in which the |A + D|/2 value of the resonator was about 0.3. In the middle of the Ho:YLF crystal, the radius of the TEM_00_ mode was about 270 μm, and the radius of the TEM_00_ mode at the position GDY-SA was about 150 μm. By the transmission of 3% OC, the maximum output power of the passively Q-switched (PQS) operation is 443 mW with an absorbed pump power of 2.4 W. The slope efficiency is 36.1% (Figure 4). With an insert displaying the fluctuation in maximum output power over one hour, the instability of the average output powers were approximately 4.1% and 3.4% of the two output couplers OCs (Figure 5). At the same time, we took measurements of the emission spectrum using a spectrometer (SOL-MS3504i, SOL-instrument Ltd., Minsk, Republic of Belarus) with a wavelength resolution of 0.34 nm. The illustration in Figure 5 shows that the pulsed laser emission spectra were centered at 2063.38 and 2062.07 nm, respectively.

**Figure 6:**
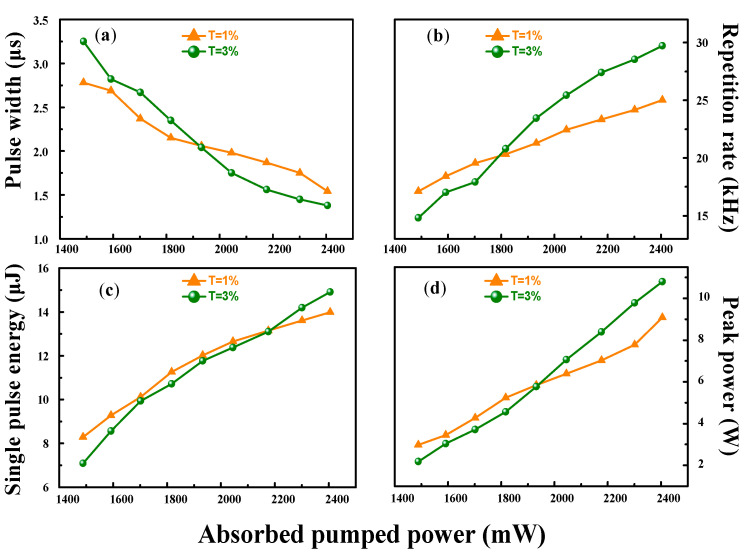
(**a**) Pulse widths, (**b**) repetition rates, (**c**) single pulse energies, and (**d**) peak powers as functions of the absorbed pump power.

In Figure 6 the repetition rate, pulse duration, peak power, and single-pulse energy dependence results of the PQS laser are plotted for different transmissions. Combining the four sets of graphs, we can see an obvious pattern. When the absorbed pump power increases, the repetition rates, peak powers and single pulse energies gradually increase, while the pulse widths become smaller. Using T = 3% OC, the shortest pulse width was 1.38 μs, and the highest pulse repetition rate was 29.72 kHz, corresponding to a pulse energy of 14.91 μJ and a peak power of 10.8 W. Table 1 summarizes the comparison results of different OC mirrors with transmissions of 1% and 3%.

**Figure 7:**
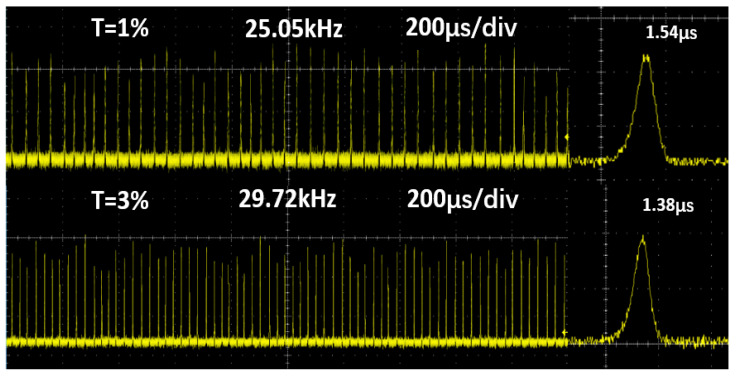
Oscilloscope display of PQS pulse trains at the maximum output power.

**Figure 8:**
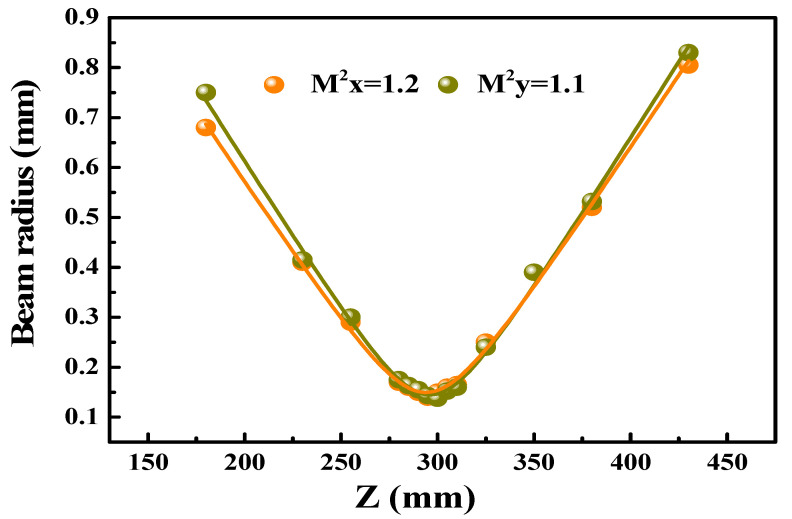
Beam quality.

Figure 7 gives a description of the typical pulse trains at the maximum average output power captured by the digital oscilloscope (MDO4104C, Tektronix, Beaverton, OR, USA) with 1 GHz and a fast photodiode detector (ET-5000, Electro-Optics, Traverse City, MI, USA). Finally, we gauged spatial beam profile (M^2^) of Q-switched Ho:YLF laser with T = 3% OC in the highest output level using the 90/10 knife-edge way. We calculated that the horizontal and vertical values are about 1.2 and 1.1 respectively, as shown in Figure 8.

## 5. Conclusions

In conclusion, GDY nanomaterial was successfully preparative using the liquid/liquid interface method and as a SA employed in Ho pulsed laser around 2.1 μm. A maximum average output power of 443 mW was obtained, corresponding to a pulse energy of 14.91 nJ and a peak power of 10.8 W. For all we know, this is the first report presenting a GDY-based Q-switched Ho-doped laser by Tm: Fiber laser pump. The optical modulator of GDY material has simple preparation process, low cost, flexible design, and is expected to be commercialized. In the future, by optimizing the parameters of the GDY-SA and designing resonator, ultrafast laser will promising be obtained near the 2.1 μm mid-infrared region.

## Figures and Tables

**Table 1:** Performances of the PQS Ho: YLF laser under different OCs.

Transmittance of the OC	T = 1%	T = 3%
Shortest pulse width/µs	1.54	1.38
Repetition rate/kHz	25.02	29.72
Peak power/W	9.08	10.8
Single pulse energy/µJ	13.99	14.91
